# The Relationship Between Unexplained Chest Pain in Children and Head-Up Tilt Test

**DOI:** 10.3389/fped.2022.901919

**Published:** 2022-06-02

**Authors:** Ying Wang, Shuo Wang, Runmei Zou, Siyang Chen, Fang Li, Yuwen Wang, Yi Xu, Cheng Wang

**Affiliations:** ^1^Department of Pediatric Cardiovasology, Children’s Medical Center, The Second Xiangya Hospital, Central South University, Changsha, China; ^2^Department of Pharmacy, Institute of Clinical Pharmacy, The Second Xiangya Hospital, Central South University, Changsha, China; ^3^Department of Neonatology, Xiangya Hospital, Central South University, Changsha, China; ^4^The Affiliated Hospital of Xiangnan University, Chenzhou, China

**Keywords:** unexplained chest pain, children, head-up tilt test, autonomic nervous function, neurally mediated syncope

## Abstract

**Background:**

To explore the relationship between unexplained chest pain in children and head-up tilt test (HUTT).

**Materials and Methods:**

A total of 825 children with the main symptom of unexplained chest pain were admitted to the Specialist Outpatient Clinic of Children’s Cardiovascular Disease from October 2000 to November 2021 at The Second Xiangya Hospital, Central South University. Among them, 473 were male and 352 were female, with a mean age of 10.61 ± 2.21 years. The control group included 58 cases, comprising 35 males and 23 females, with a mean age of 10.26 ± 2.66 years. The detailed history, physical examinations, conventional 12-lead electrocardiogram, chest X-ray, echocardiography, myocardial enzymes, electroencephalogram, and blood series were all examined. Disorders of the chest wall, lung, heart, mediastinum, and esophageal reflux, as well as drug effects, were ruled out. All the children underwent HUTT. Demographic description, univariate analysis, and multivariate logistic regression analysis were used to explore the possible linear or non-linear relationships between the children’s unexplained chest pain and HUTT.

**Results:**

Among the 825 chest pain cases, 301 (36.48%) were HUTT positive and 524 (63.52%) were HUTT negative. HUTT-positive patients were older than HUTT-negative patients (11.04 ± 2.03 vs. 10.34 ± 2.31 years, *P* < 0.001). The logistic regression results showed that each year of age increased the probability of being HUTT positive by 17.90% (*P* < 0.000), and females were 91.30% more likely to be HUTT positive than males (*P* < 0.000).

**Conclusion:**

Clinically unexplained chest pain in children is mainly caused by unbalanced autonomic nervous function. HUTT can help clear the cause of unexplained chest pain.

## Introduction

Chest pain is a common clinical reason for seeking medical attention. It can be seen in various diseases and is one of the 14 most common symptoms in primary care (1). Its lifetime incidence among the population is 24.6% (1). More than 650,000 pediatric patients (aged 10–21 years) present with chest pain each year, accounting for 5.2% of all cardiology consultations in inpatient and emergency departments and 15% of all outpatient visits (2). Unlike adults, chest pain in children is usually not caused by a severe medical condition (3). The causes of chest pain in pediatric patients vary, most of which can be classified as musculoskeletal, pulmonary, gastrointestinal, psychological, or cardiac (4). The proportion of chest pain caused by pediatric heart disease is very low, less than 5% (5). Due to the etiologies of chest pain, different treatment methods, improper clinical treatment, or delays in treatment can have serious consequences (6).

As the exact cause of chest pain is unknown, medical institutions often conduct a comprehensive evaluation of the patient. Treatment of these patients can involve extensive screening, medical care, and hospitalization, which is expensive and unnecessary in many cases (5). Most of these patients have a normal chest X-ray, electrocardiogram, echocardiography, and myocardial enzyme spectrum, but they complain of recurrent spontaneous chest pain. Chest pain in most school-aged children is of unknown cause. Repeated chest pain affects children’s quality of life and academic performance, and their families are profoundly troubled and visit doctors frequently, contributing to patients’ medical costs and frustration (7).

The evaluation of chest pain in children relies heavily on a detailed history and physical examination, and most studies fail to identify the cause. Early identification of the possible causes of chest pain has attracted widespread attention from medical workers. At present, the cause of chest tightness and pain in children reported in China is mostly caused by autonomic dysfunction. Cardiac autonomic function status can be assessed by head-up tilt test (HUTT), postural changes, heart rate variability and other methods. HUTT is an important method to evaluate autonomic nerve function clinically (8, 9). At present, HUTT has become the gold standard for diagnosing vasovagal syncope (VVS). It can induce syncope or the recurrence of syncope premonitory caused by an imbalance of autonomic nerve function through passive body position changes (10, 11). Kumar et al. (12) studied 25 children with unexplained chest pain and 36 children with unexplained dizziness in 2009. Both groups underwent HUTT examination. The results showed no significant difference in the composition ratio of HUTT response types between the two groups (*P* > 0.05). Therefore, unexplained chest pain in children may be closely related to autonomic disorders. As for children with unexplained chest pain without organic cardiovascular disease, a timely HUTT can contribute to the diagnosis of the cause. In the present study, HUTT was performed on children with unexplained chest pain and compared to healthy volunteers to explore the relationship between unexplained chest pain and HUTT in children.

## Materials and Methods

### Study Population

This work is a retrospectively reviewed study aiming to explore the relationship between unexplained chest pain and HUTT results. We examined 4- to 15-year-old children (*n* = 825) who had chest pain or distress of unknown cause from the Children’s Medical Center, The Second Xiangya Hospital, Central South University, from October 2000 to November 2021. Healthy control children were also enrolled (*n* = 58, aged 4–15 years old) and given a health examination at the hospital at the same time for comparison.

### Inclusion and Exclusion Criteria

**FIGURE 1:**
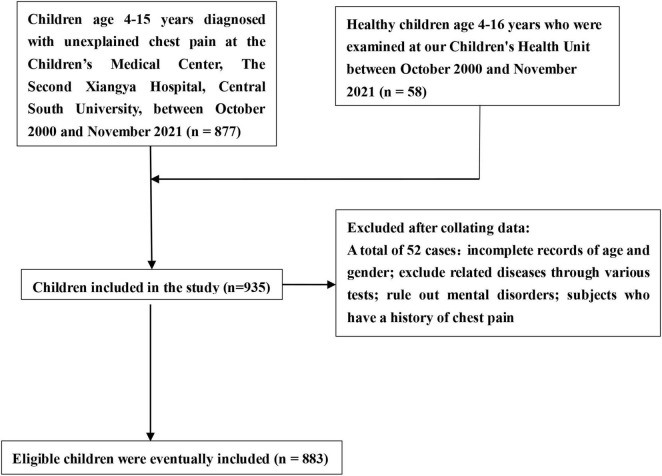
Inclusion and exclusion criteria.

Children who presented to the Children’s Medical Center with symptoms of chest pain were included in the study group. None of the children in the control group had symptoms of chest pain. Chest wall, lung, mediastinal, esophageal reflux, and cardiac diseases were excluded by assessing history, physical examination, baseline laboratory tests, 12-lead electrocardiogram (ECG), echocardiography, chest radiograph, pulmonary function, and exhaled nitric oxide. A clinical assessment by an experienced psychologist ruled out psychogenic disorders, incomplete data, and a previous history of chest pain. All subjects underwent HUTT according to clinical guidelines (13) (Figure 1).

### Head-Up Tilt Test Protocol

Head-up tilt test consists of two stages: basic HUTT (BHUT) and sublingual nitroglycerin provoked HUTT (SNHUT). The protocol was carried out in accordance with previous research (1). HUTT was approved by the Ethics Committee of The Second Xiangya Hospital, Central South University. Informed consent was issued by all subjects directly or their guardians. HUT-821 tilt test monitoring system (Beijing Juchi Medical Technology Co., Ltd.) and SHUT-100 tilt test monitoring software system (Beijing Standley Technology Co., Ltd). The child was placed on the tilt bed for around 10 min, with the bands fixed to avoid bending the legs. The basic heart rate (HR), blood pressure (BP), and baseline ECG were recorded during this period. Then, the bed was tilted upward by 60°. HR, BP, and ECG were monitored simultaneously and continuously until either the 45-min duration was up or the development of syncope or intolerable near-syncope symptoms. The child would rapidly be placed in the supine position if syncope occurred. If syncope or pre-syncope symptoms did not happen, the child would remain in the same position for 20 min after sublingual nitroglycerin administration of 4–6 μg/kg (maximum ≤300 μg). The slanted posture was maintained, and HR, BP, and ECG were monitored until syncope or pre-syncope occurred after 20 min.

### Clinical Diagnosis

In the study, VVS, postural tachycardia syndrome (POTS), orthostatic hypotension (OH), and orthostatic hypertension (OHT) were all positive for HUTT. VVS was defined as the development of syncope or pre-syncope accompanied by hypotension: (1) systolic BP (SBP) ≤80 mmHg or diastolic BP (DBP) ≤50 mmHg or mean pressure decrease ≥25%; (2) HR <75 bpm for children 4–6 years old, <65 bpm for children 6–8 years old, and <60 bpm for children older than 8 years; (3) ECG showing sinus arrest and premature junctional contractions; and (4) atrioventricular block and cardiac arrest ≥3 s. VVS was divided into three types of reactions: vasoinhibitory type (VVS-VI, significant reduction in BP but insignificant change in HR), cardioinhibitory type (VVS-CI, significant reduction in HR but insignificant change in BP), and mixed type (VVS-M, significant reduction both in BP and HR).

Postural tachycardia syndrome was defined as dizziness, chest distress, headache, palpitation, and pallor with one of the following within 10 min of HUTT: an increase in HR ≥40 bpm in children and adolescents or by a maximum HR >130 bpm in children 4–12 years old and >125 bpm in adolescents 12–18 years old.

Orthostatic hypotension was defined as orthostatic intolerance symptoms and a decrease in systolic BP ≥20 mmHg and/or diastolic BP ≥10 mmHg within 3 min of HUTT.

Orthostatic hypertension was characterized as follows: supine BP is normal and during the initial 3 min of HUTT or the standing test, and SBP increases ≥20 mmHg and/or DBP increases ≥25 mmHg (in children 4–12 years old) or ≥20 mmHg (in adolescents 12–18 years old) from supine to upright or upright BP ≥130/90 mmHg (in children 4–12 years old) or ≥140/90 mmHg (in adolescents 12–18 years old) without a noticeable change in HR.

### Statistical Analysis

Continuous variables were normally distributed. The data were described as the mean ± standard deviation and analyzed using a *t*-test. For non-normally distributed data, continuous variables were expressed as the median of the interquartile range and analyzed using the Mann–Whitney *U* test. Dichotomous variables were expressed as percentages and compared using the χ*^2^* test or Fisher’s exact test. We used multiple logistic regression to analyze the possible association between the HUTT results and age and sex. Two models were constructed to illustrate the stability of this relationship: Model I was unadjusted, and Model II was adjusted for sex and age. All the analyses were performed with the statistical software packages R (version 3.4.3; The R Foundation)^1^, EmpowerStats (X&Y Solutions, Inc., Boston, MA, United States)^2^, and SPSS 23 (IBM Corp., Armonk, NY, United States). *P*-values < 0.05 (two-sided) were considered statistically significant.

## Results

### Clinical Characteristics and Head-Up Tilt Test Positive Rate of the Study and Control Groups [*n* = 883, Mean ± SD, *n* (%)]

No statistical differences were found between the two groups in terms of age and sex (*P* > 0.05). The positive rate was significant between the two groups. 36.48% of patients with chest pain had a positive response to HUTT, which was higher than that of healthy individuals (36.48% vs. 5.17%, respectively, *P* = 0.000) (Table 1).

**TABLE 1:** Clinical characteristics and HUTT positive rate of the study and control groups [*n* = 883, mean ± SD, *n* (%)].

	Control group	Study group	*t/*χ^2^ value	*P*-value
**Sex**				
Male	35 (60.34)	473 (57.33)	0.097	0.755
Female	23 (39.66)	352 (42.67)		
Age (year)	10.26 ± 2.66	10.61 ± 2.21	−0.954	0.344
HUTT positive	3 (5.17)	301 (36.48)	23.536	0.000
HUTT negative	55 (94.83)	524 (63.52)		
				

### Clinical Data Comparison Between Chest Pain Patients With Positive and Negative Responses to Head-Up Tilt Test

In the study group, a significant difference was found in the proportion of males and females who had a positive response to HUTT (*P* < 0.001), and the average age was older (11.04 ± 2.03 vs. 10.34 ± 2.31 years, respectively, *P* < 0.000) (Table 2).

**TABLE 2:** Clinical data comparison between patients with positive and negative HUTT response in the study group [mean ± SD, *n* (%)].

HUTT result	HUTT negative	HUTT positive	*P-*value
*n*	579	304	
Age (year)	10.34 ± 2.31	11.04 ± 2.03	<0.001
**Sex**			<0.001
Male	360 (62.18)	148 (48.68)	
Female	219 (37.82)	156 (51.32)	

### Univariate Analysis of Age and Sex in Head-Up Tilt Test-Positive Results

Through univariate logistic regression analysis, we found that age and sex could be risk factors for HUTT results. For each 1-year increase in age, the probability of a HUTT-positive result increased by 15.50% (*P* < 0.000). Females were more likely than males to have a positive attitude toward HUTT, increasing the probability of being HUTT positive by 73.30% (*P* < 0.000) (Table 3).

**TABLE 3:** Univariate analysis of age and sex on HUTT-positive results [*n* = 883, mean ± SD, *n* (%)].

Exposure	Statistics	OR (95% CI)	*P*-value
Age (year)	10.58 ± 2.24	1.155 (1.083, 1.232)	<0.000
**Sex**			
Male	508 (57.50)	1.00	
Female	375 (42.50)	1.733 (1.309, 2.294)	0.000

### The Relationship Between Age and Head-Up Tilt Test Results and the Relationship Between Sex and Head-Up Tilt Test in Different Models

After accounting for the confounding factor of sex, the independent effect of age on HUTT results remained stable, with each additional year of age increasing the likelihood of a positive HUTT 17.90% (*P* < 0.000). After accounting for age as a confounding factor, the independent effect of sex on the HUTT results remained stable, and females were 91.30% more likely to be HUTT positive than males (*P* < 0.000) (Table 4).

**TABLE 4:** The relationship between age and HUTT results and the relationship between sex and HUTT in different models.

	*Sex*		*Age*	
	OR (95% CI)	*P*-value	OR (95% CI)	*P*-value
Model I	1.733 (1.309, 2.294)	0.0001	1.155 (1.083, 1.232)	<0.000
Model II	1.913 (1.433, 2.553)	<0.0001	1.179 (1.103, 1.259)	<0.000

*Data in the table: OR (95% CI), P-value.*

*Result variable: HUTT results.*

*Exposure variable: Sex/Age.*

*Model I in sex adjusted for: None.*

*Model II in sex adjusted for: Age.*

*Model I in age adjusted for: None.*

*Model II in age adjusted for: Sex.*

### Head-Up Tilt Test Results for Each Group and Hemodynamic Type

**FIGURE 2:**
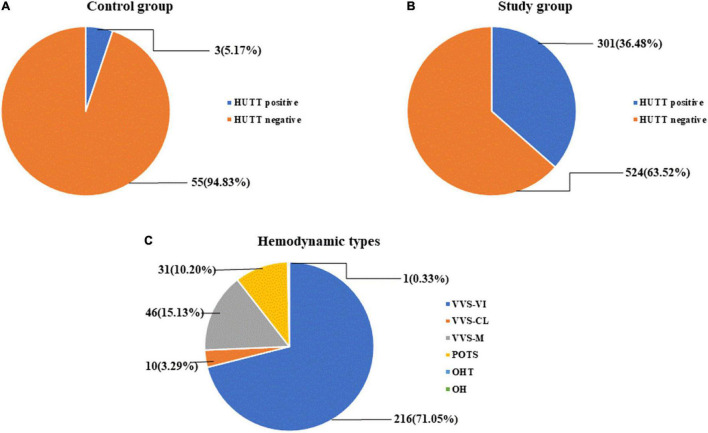
HUTT results for each group and hemodynamic type [*n* (%)]. **(A)** HUTT results of the control group. **(B)** HUTT results of the study group. **(C)** Hemodynamic type in HUTT.

In the study group, 301 (36.48%) were HUTT positive, and 524 (63.52%) were negative; in the control group, 3 (5.17%) were HUTT positive and 55 (94.83%) were negative. Among the 883 subjects, 304 cases (34.43%) were HUTT positive, including 216 cases (71.05%) of VVS-VI, 10 cases (3.29%) of VVS-CI, 46 cases (15.13%) of VVS-M, 31 cases (10.20%) of POTS, and 1 case (0.33%) of OHT. The OH type was not found in this study (Figure 2).

## Discussion

Chest pain is a common complaint in the pediatric population, occurring in outpatient and emergency department settings (14). Most of the causes of chest pain in children are chest wall pain and lung and psychological diseases, and a small number of reasons are heart, traumatic, and gastrointestinal diseases (15). We can identify or suspect an organic cause through the patient’s history and a comprehensive physical examination. However, for most patients with chest pain of suspected non-organic cause, although auxiliary examinations are also conducted, the exact cause has not yet been identified (14). Many repeated tests tie up medical facility resources and cost families money (16).

The organic causes of unexplained chest pain are often difficult to identify, and studies have shown that mental and social factors are associated with unexplained chest pain. Eliacik et al. (9) found that 100 teens diagnosed with unexplained chest pain were more likely to be bored, anxious, and stressed and have sleep disturbances, headaches, back pain, and impaired social functioning compared to the control group. Lipsitz et al. (15) found that in 32 children diagnosed with chest pain who had a mean age of 12.8 years, 81% were diagnosed with a psychiatric disorder and 28% with a full-criteria panic disorder. Results showed significant associations between unexplained chest pain, physical symptoms, depression, and impaired emotional and social functioning. Mental and social factors could cause autonomic dysfunction, leading to symptoms such as chest pain of unknown clinical symptoms.

In addition to the influence of social and mental factors, some studies have shown that unexplained chest pain is related to sex. White et al. (17) found that the link between chest pain and alexithymia and pain was stronger in men than in women. Conversely, Fagring et al. (18) found no sex difference in the intensity of chest pain, and that sex could only describe pain differently. Our study also showed no significant difference in chest pain in terms of sex in children.

When there is diagnostic doubt and previous history and physical examination have ruled out an organic cause of chest pain, can HUTT be performed to verify that chest pain is a neurological problem? In the past, HUTT was an essential tool for assessing autonomic nervous system function, and tilt testing was first described in the early 1990s as an investigative tool for unexplained or suspected VVS (19). HUTT induces a series of autonomic dysfunction symptoms such as chest tightness, chest pain, dizziness, etc., in patients with autonomic dysfunction by stimulating the Bezold-Jarisch reflex. Unexplained chest tightness and/or chest pain are closely related to autonomic dysfunction. According to the European Syncope Guidelines, Barón-Esquivias et al. (20) used HUTT to retrospectively predict the hemodynamic classification (VVS, OHT) of 1,058 patients diagnosed with syncope. The European Syncope Guidelines recommendations combined with HUTT identified 81% of the patients as having non-cardiac syncope, thus potentially avoiding a large number of unnecessary diagnostic tests. Zou et al. (8) found that children with unexplained sighs responded positively to HUTT and that the results indicated that sighing was associated with autonomic nervous system dysfunction. HUTT is an important tool for assessing autonomic nervous system function and can also be used to describe the hemodynamic pattern of unexplained sighing in children and adolescents (13). Therefore, we believe that HUTT has potential diagnostic value for unexplained chest pain.

In this study, 825 children with unexplained chest pain were examined using HUTT, and 36.48% were found to be HUTT positive. The HUTT performance of the control group and the study group was *P* < 0.000, and a significant difference was found between the two groups. The symptoms of children with unexplained chest pain were significantly different from those of the control group. This is related to autonomic dysfunction and can be predicted by HUTT. Moreover, the HUTT-positive group was found to be older than the HUTT-negative group.

According to the analysis of the clinical characteristics of our HUTT results, sex has a significant effect on a positive HUTT result, and HUTT may be a useful auxiliary tool in assessing the hidden risk of unexplained chest pain in children. We analyzed the reasons why sex affects HUTT results. While standing, approximately 700 ml of blood is drawn from the chest into the gravity-dependent area (21). The blood pooling in the compliant area (e.g., the visceral area) is isolated from the systemic circulation, resulting in decreased venous return and reduced cardiac preload, thereby reducing cardiac output. When arterial baroreceptors are relieved, sympathetic disinhibition produces a compensatory response in which the autonomic nervous system protects blood pressure by increasing heart rate and α1-adrenergic vasoconstriction. After prolonged standing, renin, angiotensin II, aldosterone, and vasopressin maintain blood pressure by altering fluid balance/retention. If these short- and long-term compensatory mechanisms are insufficient, blood pressure and cerebral perfusion cannot be maintained, and syncope may occur (22). From the perspective of body structure, the blood volume of a female’s lower body is greater (23). When the lower body is under negative pressure, the blood volume of a female’s pelvic area increases by 83% (23). The pelvic segment may contain part of the visceral circulation. During the supine position (HUTT), females show more blood volume and less visceral vasoconstriction, leading to visceral stasis, lower blood pressure, and lower orthostatic endurance (22). Jarvis et al. (24) showed that females had a lower orthostatic tilt tolerance than males, which could be related to a smaller visceral vasoconstriction reserve. Xu et al. (25) showed that the age of syncope patients was younger than that of non-syncope patients and that the HUTT regimen had higher sensitivity in young female syncope patients. Combining the research reports and our findings, the proportion of females with positive HUTT results was greater than that of males.

Age also had an effect on the HUTT results. Metrics related to sympathetic activity, such as increased muscle sympathetic activity and norepinephrine spillover, increase with age (26). In contrast, metrics related to parasympathetic activity, such as heart rate variability (27) and cardiac vascular baroreflex sensitivity (27, 28), decreased with aging. In older subjects, heart rate responses to postural changes were attenuated, and conversely, their vascular responses were enhanced. Age-related cardiovascular autonomic dysfunction may lead to altered strategies for maintaining upright blood pressure homeostasis (29). Moreover, age increases the rate of sympathetic norepinephrine release and reduces interstitial norepinephrine circulation, which occurs in old age. Decreased sympathetic and parasympathetic components of the heart have been observed in humans (30). Autonomic control of blood pressure appeared to decline with age (31). These studies indicate that the sensitivity of cardiac β-adrenergic receptors decreases with age, a process that may lead to the impairment of autonomic function. Guo et al. (32) found that in an analysis of 175 HUTT-positive patients, age (OR value: 1.034, *P* = 0.011) was independently associated with HUTT-positive VVS syncope recurrence, and that the area under the receiving operating characteristic curve of age predicting HUTT-positive VVS patients with syncope recurrence was 0.688. The recurrence rate of syncope increased with age, especially in women. Hu et al. (33) conducted a questionnaire survey on 4,352 randomly selected children and adolescents aged 2–18 in Changsha City, and the results showed that the incidence of syncope in adolescence (28.85%) was higher in school-age (8.32%) and preschool age (2.71%) (*P* < 0.01). The incidence of syncope was higher in adolescent females than in males (31.72% vs. 26.25%, *P* < 0.05). These findings are consistent with our results.

In summary, this study showed that 36.48% of children with unexplained chest tightness and/or chest pain had HUTT positive, indicating that some children with unexplained chest tightness and/or chest pain may be closely related to autonomic dysfunction. HUTT can help clear the cause of unexplained chest pain. The probability of a positive HUTT reaction can be predicted according to the age and sex of a child. Slightly older female children with chest pain need more attention, and HUTT can be arranged as soon as possible. It is suggested that children with unexplained chest tightness and/or chest pain without organic cardiovascular disease should undergo HUTT examination in time to help clarify the etiological diagnosis.

## Data Availability Statement

The original contributions presented in this study are included in the article/supplementary material, further inquiries can be directed to the corresponding author/s.

## Ethics Statement

The studies involving human participants were reviewed and approved by the Medical Ethical Committee, The Second Xiangya Hospital, Central South University. Written informed consent to participate in this study was provided by the participants’ legal guardian/next of kin.

## Author Contributions

YW, CW, and YX conceived the study. SW, RZ, FL, and YWW collected and reviewed subjects’ data. YW, SW, and SC performed the statistical analysis. YW and SC drafted the manuscript. All authors contributed to the revision of the manuscript.

## Conflict of Interest

The authors declare that the research was conducted in the absence of any commercial or financial relationships that could be construed as a potential conflict of interest.

## Publisher’s Note

All claims expressed in this article are solely those of the authors and do not necessarily represent those of their affiliated organizations, or those of the publisher, the editors and the reviewers. Any product that may be evaluated in this article, or claim that may be made by its manufacturer, is not guaranteed or endorsed by the publisher.

## Footnotes

1
http://www.R-project.org

2
http://www.empowerstats.com
